# Some Reflections by an Ex-Patient

**Published:** 1921-06-18

**Authors:** 


### PENSIONS AND NEURASTHENIA.

### Some Reflections by an Ex-Patient.

It has been suggested that in a paper usually devoted to the views of the medical profession and the administrative class generally, some brief account of the experiences of one who was successively a private soldier, an "administered" pensioner, and an extremely subordinate official in the Ministry, might be of interest, if only as an illustration of a difference in view-points!

Such a record, from the medical point of view, should presumably start with some account of the illness leading to military discharge. In the December of '17 I "came down the line" from Passchendaele with a rather severe bout of trench fever, and, after a month at the Base, found myself in a great provincial hospital. As the fever disappeared, an extraordinarily unpleasant complaint, which I afterwards learned to call a phobia, developed, and, as I was convinced that I was going mad, and never dare tell anyone of my feelings, for some weeks I lived in a little inferno. The civilian doctor in charge of the ward at last recognised the symptoms, reassured me of my sanity, treated me with some compound of hyoscin—and I slowly began to feel better.

There were neurological wards in the hospital, but I was never removed to them, nor was my official description ever changed—I strongly suspected because my doctor found my case interesting and wished to keep me to himself. From my point of view this led to trouble, for in the mid-April, when the Push was sending over thousands daily, a high officer of the R.A.M.C. happened to inspect my ward, found a bed occupied for months by a man labelled only trench fever, and with some indignant remarks on malingerers—I enlisted in '14, while he, as I took pains to find out, had never left his own town—bundled me out. A fortnight later the phobia reappeared stronger than ever, and since there could now be no possible doubt about it, I was labelled neurasthenia, and marked for discharge.

For some reason this did not materialise for a couple of months, and for that time I drifted about a huge camp, excused all duties and parades, a constant source of friction to my N.C.O.s, of jealousy to my mates, a misery to myself, and steadily getting worse all the time. I was supposed at certain hours to attend the M.O.'s orderly to drink bromide, but as it had no effect I did not go. I was eventually recommended for immediate treatment in a Pensions hospital, and after ten days spent at home with my wife I passed into a Pensions neurological hospital. My first impressions were, frankly, of sheer horror at my fellow-patients.

A week or so later I drifted into the position of office boy to the hospital; in another week or so I *was* to all intents and purposes the office, "patient clerk," M.O.'s secretary—everything, though all entirely unofficially. Then I found out all about the treatment, and *got cured!* In a medical paper it would be impertinent for a layman to discuss psycho-therapy. All I can say is that it undoubtedly cured me of an exceptionally bad phobia; that two years later I am *still cured!* In the admittance of newcomers I broke miles of red-tape, but identifying myself with what I thought were my master's interests, I used deliberately to select what I considered the best—*i.e.*, the most likely to be cured patients from the enormous lists sent in almost daily. Realising the futility of turning out the average neurasthenic without a job immediately to go to, and learning by experience that the Ministry Training Schemes involved months of delay, I used to pass over men in the lists with such descriptions as "ex-warrant officer, Regular Army," or "casual labourer." Men I saw had been in and out of hospitals for months I avoided, as also "shell-shocks" with no overseas service. In fact, as far as I could I wanted the man who seemed likely to be better off cured! All this was, of course, highly irregular. As an ex-Tommy "patient clerk" I was scarcely in a position to alter the system, or lack of system, with which the Ministry treated neurasthenics, but in a very tiny way I could look after the interests of that particular hospital, and I did.

Had I been a high official I think I should have got rid of the nurses. Not that I disliked them; we were the best of friends. I suppose the public likes to think of them as ministering angels nursing the wounded heroes; only, as no nursing was needed, and a couple of ex-Metropolitan policemen would have been far more efficacious as door-keepers, and less costly, it seemed rather a costly camouflage!

The public, by the way, must have been extremely bad for us. When I was admitted in '18, before the Armistice, we were still heroes, and welcome guests in a good many houses where as civilians we should never have gained admission. The natural result was a good deal of what I suppose the doctor calls "subconscious malingering." As a patient myself I used to hear both sides of the story.

The pension, however, was not enough, not nearly enough, for my own wants, and so—against my doctor's wishes, since I had no work to go out to—I insisted on leaving in February 1919.

Sending my wife to a relative's till such time as I could start again, I took the very first job I could find at any money, oddly enough a minor clerkship in a pensions office in a poor district of London. At half-past nine I fought my way through a surging mob of pensioners and would-be pensioners. As fast as the crowds could be marshalled, the dossiers were brought upstairs. Picking up one at random—there were four of us in the room, all "interviewing" at once—I shouted the name on it. I had then an average of five minutes in which to go through my client's papers, and a genuine "pensioneer" would have a pile several inches thick, advise him, help him to fill up some dozens of forms, and get him out feeling that he had met a really sympathetic official, and that at last his case was going to be attended to! This continued till about two; after lunch I had to write letters on the cases I had dealt with, and generally tidy up my mess. I finished about six, except once a week when the office was open till ten.

I thoroughly enjoyed my job, with the exception of my pay, which was £3 3s. 6d. a week, less than many of the men who came to see me were drawing out of pensions and allowances. But for the rest of my life I shall never allow myself to be called a neurasthenic!

Neurasthenia was the stock disease for those who could think of nothing better, and after my experiences as a pensions clerk, little pride in the name is left. The majority of the genuine cases had their pensions fixed, and only needed our help when something went wrong in the issue of their pensions. Most of my own customers were those demobilised as fit, and who, failing to find a job, put in claims really as a sort of out-of-work benefit, and they almost invariably claimed on neurasthenia!

